# Supplementary material for: DNAJA2 deficiency activates cGAS-STING pathway via the induction of aberrant mitosis and chromosome instability
Source: Nat Commun. 2023 Aug 28;14:5246. doi: 10.1038/s41467-023-40952-0 (PMC10462666; doi:10.1038/s41467-023-40952-0)
Supplement: Supplementary file 1 — Supplementary Information [file 41467_2023_40952_MOESM1_ESM.pdf]

Supplementary Information

DNAJA2 deficiency activates cGAS-STING pathway via the induction of aberrant mitosis and chromosome instability

Yaping Huang[1,6], Changzheng Lu[2,3,6], Hanzhi Wang[1], Liya Gu[1], Yang-Xin Fu[2,4*], and Guo-Min Li[1,5*]

[1]Department of Radiation Oncology, University of Texas Southwestern Medical Center, Dallas, TX, USA

[2]Department of Pathology, University of Texas Southwestern Medical Center, Dallas, TX, USA

[3]Institute of Cancer Research, Shenzhen Bay Laboratory, Shenzhen, China

[4]Department of Basic Medical Sciences, Tsinghua University of School of Medicine, Beijing, China

[5]Chinese Institutes for Medical Research, Beijing, China

[6]These authors contributed equally

***Correspondence to:**
Yang-Xin Fu (Yang-Xin.Fu@utsouthwestern.edu)
Guo-Min Li (Guo-Min.Li@UTSouthwestern.edu or gmli@cimrbj.ac.cn)

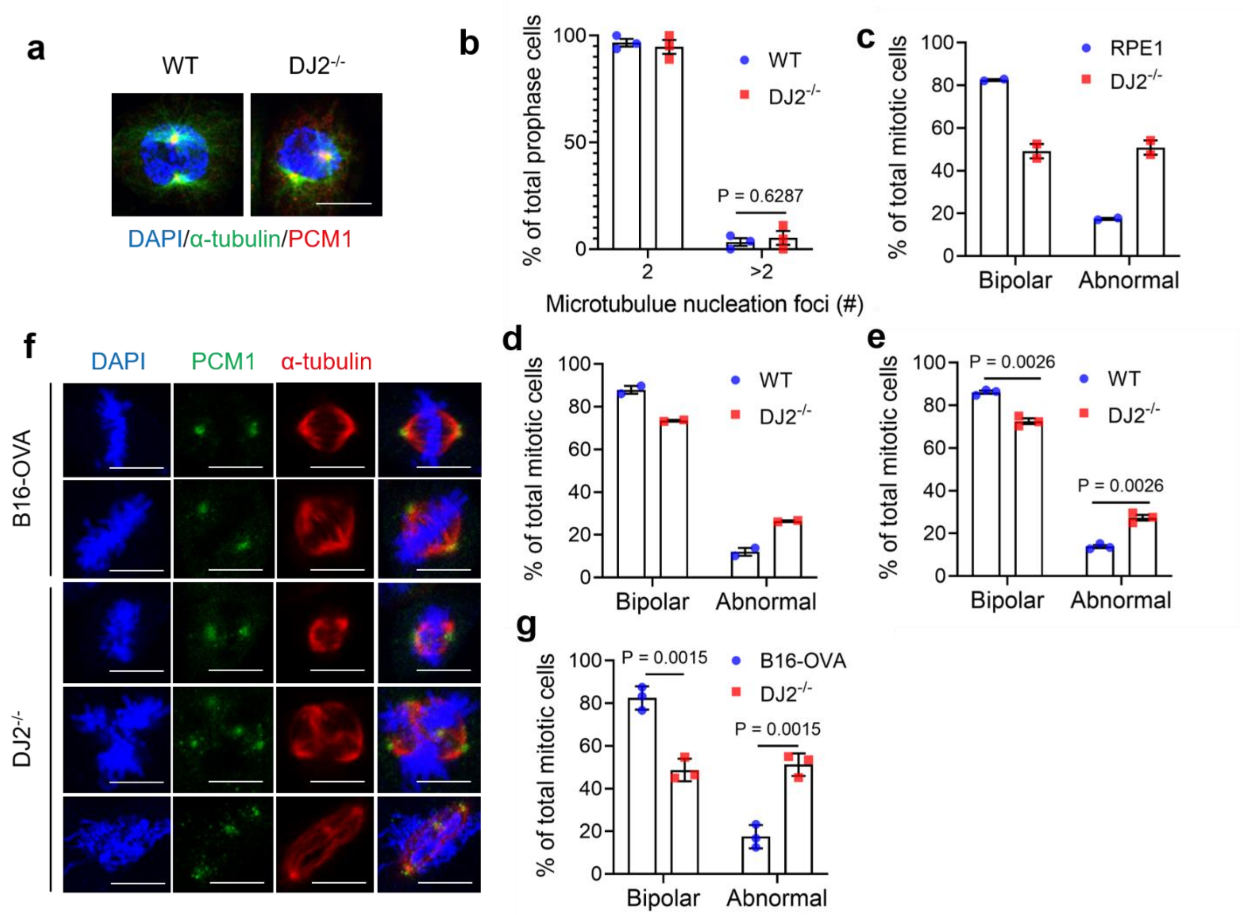

Supplementary Fig. 1: DNAJA2 deficiency causes aberrant mitosis. (**a, b**) Representative images and quantification of prophase cells with 2 well-defined centrosomes (normal) or > 2 α-tubulin/PCM1 foci (abnormal), *n* = 3. (**c-e**) Quantifications of cells with bipolar and abnormal spindles in control and $DJ2^{-/-}$ RPE1 cells (*n* = 2) (**c**), H460 cells (*n* = 2) (**d**) and SW620 cells (*n* = 3) (**e**). (**f**) Representative images showing bipolar and abnormal spindles in control and $DJ2^{-/-}$ B16-OVA cells. Scale bar, 20 μm. (**g**) Quantification of cells with bipolar and abnormal spindles in control and $DJ2^{-/-}$ B16-OVA cells, as shown in **f**, *n* = 3. Data are shown as means ± SEM of *n* experimental repeats. P values were determined by two-tailed unpaired t test with Welch's correction. Source data are provided as a Source Data file.

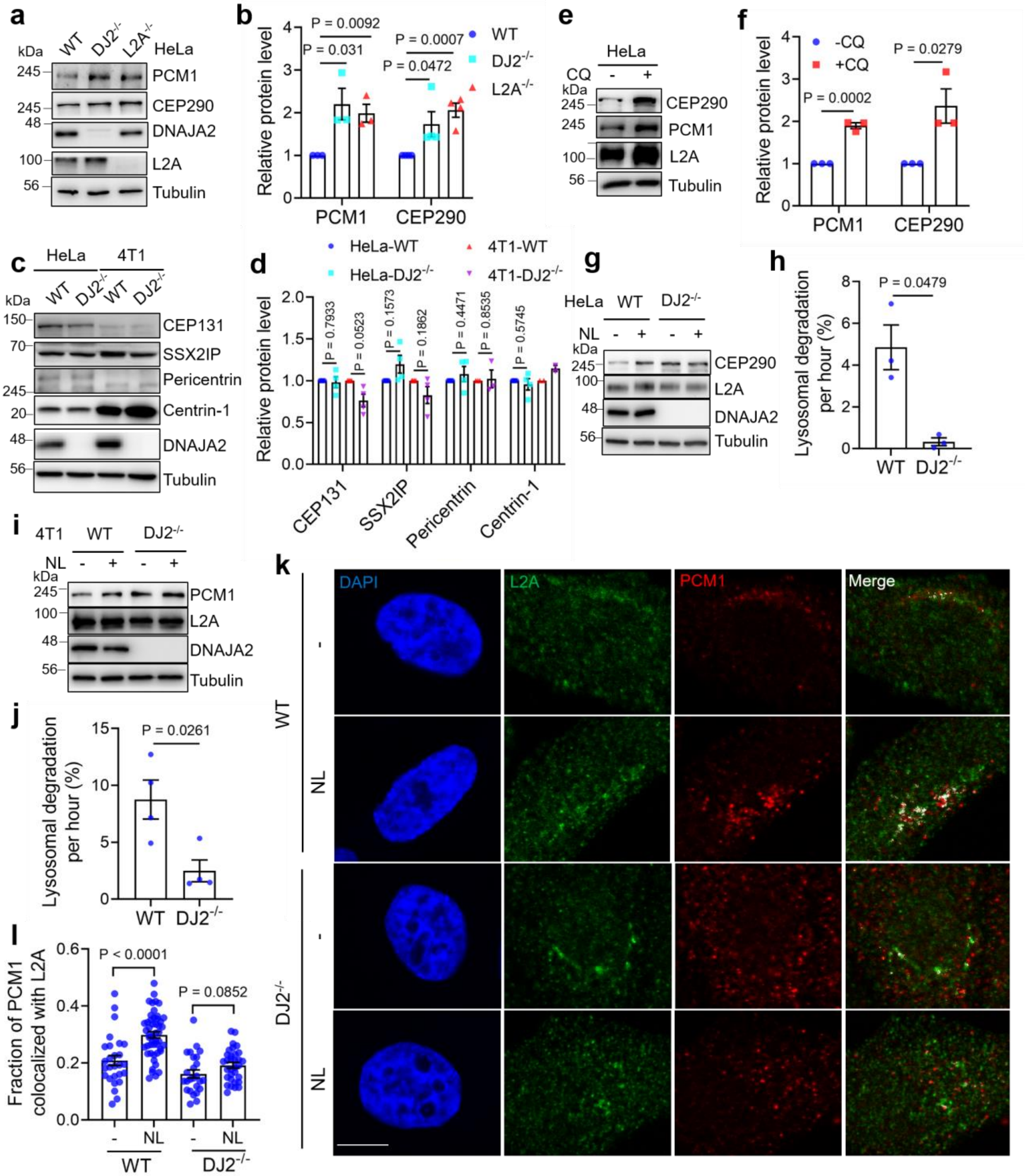

Supplementary Fig. 2: DNAJA2 regulates centriolar satellite proteolysis through chaperone-mediated autophagy. (**a**) Western blots showing elevated expression of PCM1 and CEP290 in *DJ2*$^{-/-}$ and *L2A*$^{-/-}$ HeLa cells, as compared with WT controls. (**b**) Quantifications of relative PCM1 (n = 3) and CEP290 (n = 4) levels in WT, *DJ2*$^{-/-}$ and *L2A*$^{-/-}$ HeLa cells, as shown in **a**. (**c**) Western blots showing expression levels of CEP131, SSX2IP, Pericentrin and Centrin 1

proteins in WT and *DJ2*$^{-/-}$ HeLa and 4T1 cells. (**d**) Quantifications of relative protein levels, as shown in **c**, *n* = 2 for Centrin-1 and *n* = 3 for Pericentrin in 4T1 cells, and *n* = 4 for all the others. (**e**) Western blotting analysis showing the expression levels of PCM1 and CEP290 in HeLa cells treated with or without 20 μM CQ for 16 hours. (**f**) Quantifications of relative protein levels, as shown in **e**, *n* = 3. (**g**) Western blotting analysis showing CEP290 protein level in WT and *DJ2*$^{-/-}$ HeLa cells treated with or without NL (20 mM ammonium chloride and 100 μM leupeptin) for 16h. (**h**) Calculation of CEP290 degradation rate in lysosomes in WT and *DJ2*$^{-/-}$ HeLa cells as shown in **g**, *n* = 3. (**i**) Western blotting analysis showing PCM1 protein level in WT and *DJ2*$^{-/-}$ 4T1 cells treated with or without NL for 8h. (**j**) Calculation of PCM1 degradation rate in lysosomes in WT and *DJ2*$^{-/-}$ 4T1 cells as shown in **i**, *n* = 4. (**k**) Representative images showing PCM1-LAMP2A colocalization (indicated by white dots in the merge section) in control and *DJ2*$^{-/-}$ HeLa cells treated with or without NL for 16h. Scale bar, 10 μm. (**l**) Quantification of PCM1 colocalized with LAMP2A in control (*n* = 27, 49 cells for untreated and treated) and *DJ2*$^{-/-}$ (*n* = 25, 32 cells for untreated and treated) HeLa cells, as shown in **k**. Data are shown as means ± SEM of *n* experimental repeats. The P values in figures **b** and **f** were determined by two-tailed unpaired t test and the others were determined by two-tailed unpaired t test with Welch's correction. Source data are provided as a Source Data file.

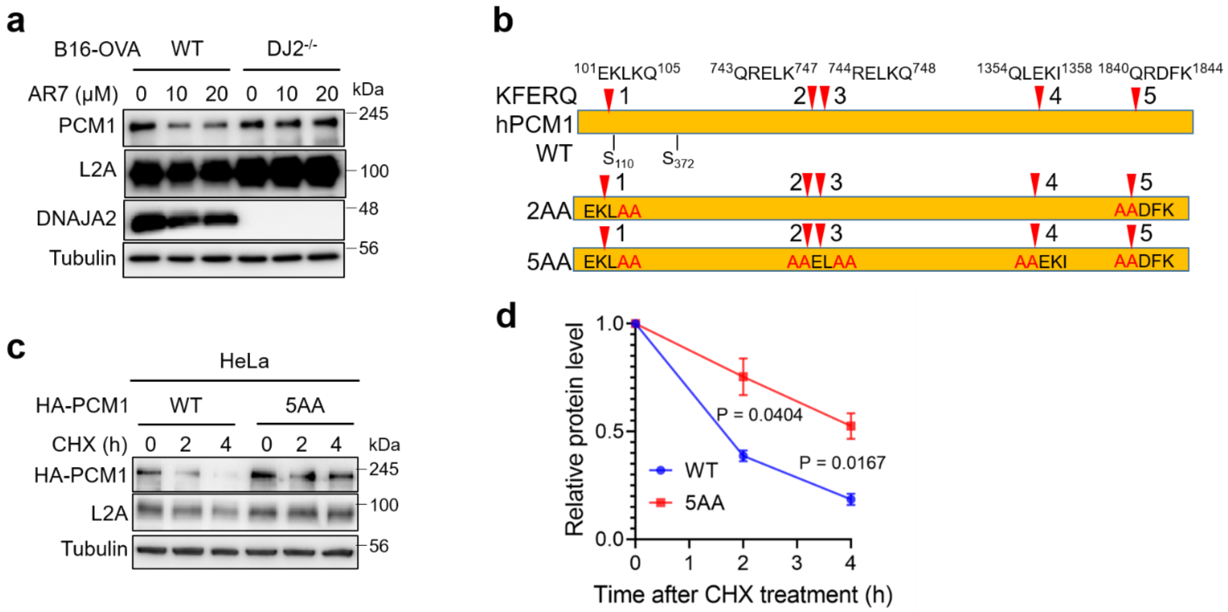

Supplementary Fig. 3: Validation of PCM1 as a CMA substrate. (**a**) Western blot analysis showing PCM1 degradation in WT and *DJ2*$^{-/-}$ B16-OVA cells treated with 10 or 20 μM AR7 for 16 hours, as indicated, *n* = 2 experimental repeats. (**b**) Schematic diagram showing locations and amino acid sequences of five canonical KFERQ-like motifs (red triangles) in human PCM1. Two serine residues phosphorylated by PLK1 and PLK4 are also highlighted. The PCM1 mutants used in this study contains RQ>AA mutations shown by red characters. (**c**) Western blots showing protein stability of WT and 5AA mutant HA-PCM1 in HeLa cells. (**d**) Quantifications of relative protein levels for WT and mutant PCM1 as shown in **c**, *n* = 3. The relative protein level was compared with the amount of the protein in time 0 after normalization with the loading control, tubulin. Data are shown as means ± SEM of *n* experimental repeats. P values were determined by two-tailed unpaired t test with Welch's correction. Source data are provided as a Source Data file.

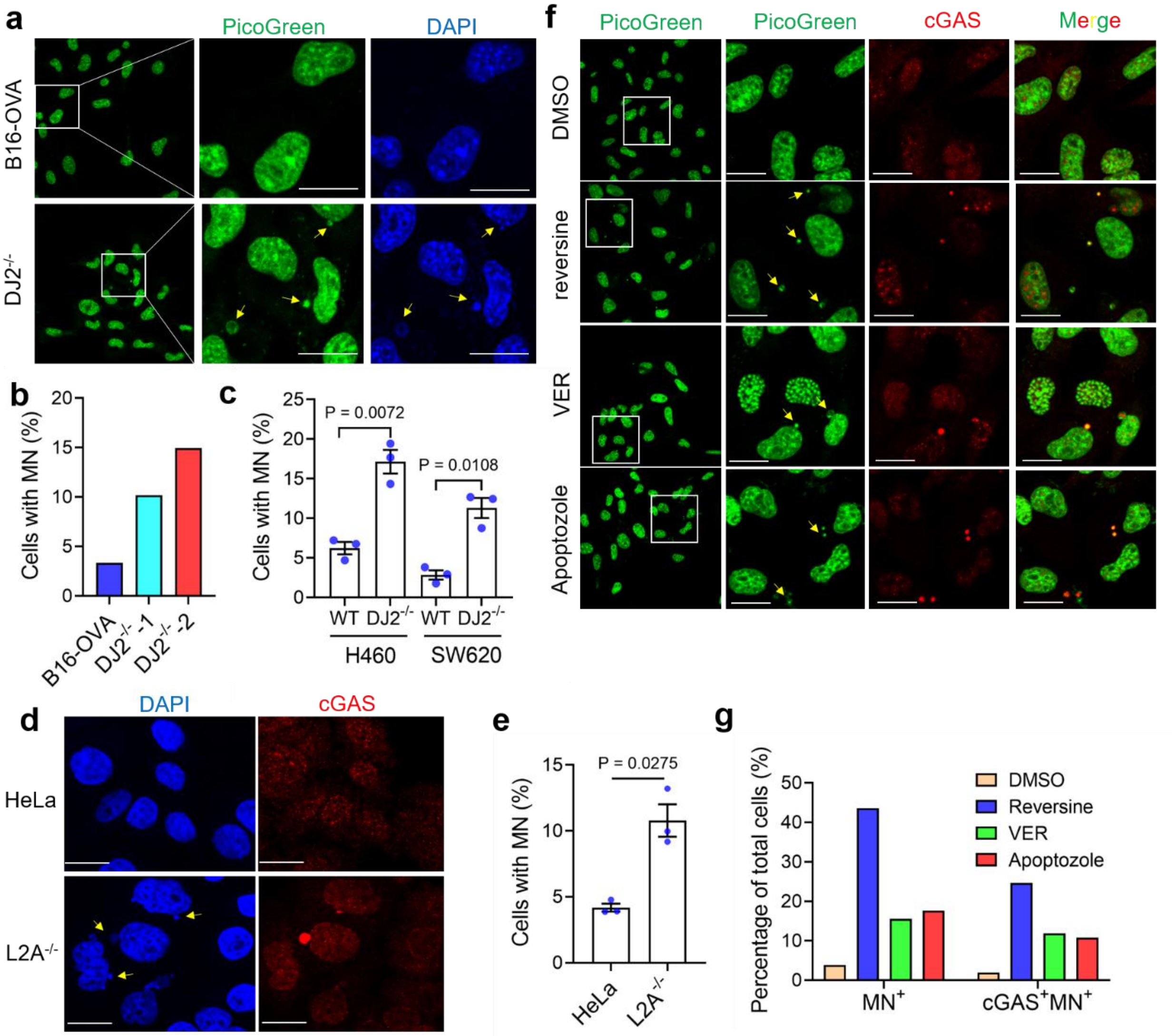

Supplementary Fig. 4: Deficiency in DNAJA2/HSC70-mediated CMA induces micronuclei. (**a**) Representative images showing micronuclei (MN, indicated by the yellow arrows) in control and *DJ2*$^{-/-}$ B16-OVA cells. (**b, c**) Quantifications of cells exhibiting MN in control and *DJ2*$^{-/-}$ B16-OVA cells (n = 1) (**b**), H460 cells and SW620 cells (n = 3) (**c**). (**d**) Representative images showing MN in control and *L2A*$^{-/-}$ HeLa cells. (**e**) Quantification of cells displaying MN in control and *L2A*$^{-/-}$ HeLa cells, as shown in **d**, n = 3. (**f**) Representative images showing MN (yellow arrows) in B16-OVA cells treated with DMSO, 0.5 μM reversine, 10 μM VER-155008 (VER) or 10 μM Apoptozole for 24 hours, as indicated. (**g**) Quantifications of MN-positive (MN^+) and cGAS/MN-positive ($cGAS^+MN^+$) cells in B16-OVA cells treated with the indicated chemicals, as shown in **f**, n = 1. Scale bar, 20 μm. Data are shown as means ± SEM of n experimental repeats. P values were determined by two-tailed unpaired t test with Welch's correction. Source data are provided as a Source Data file.

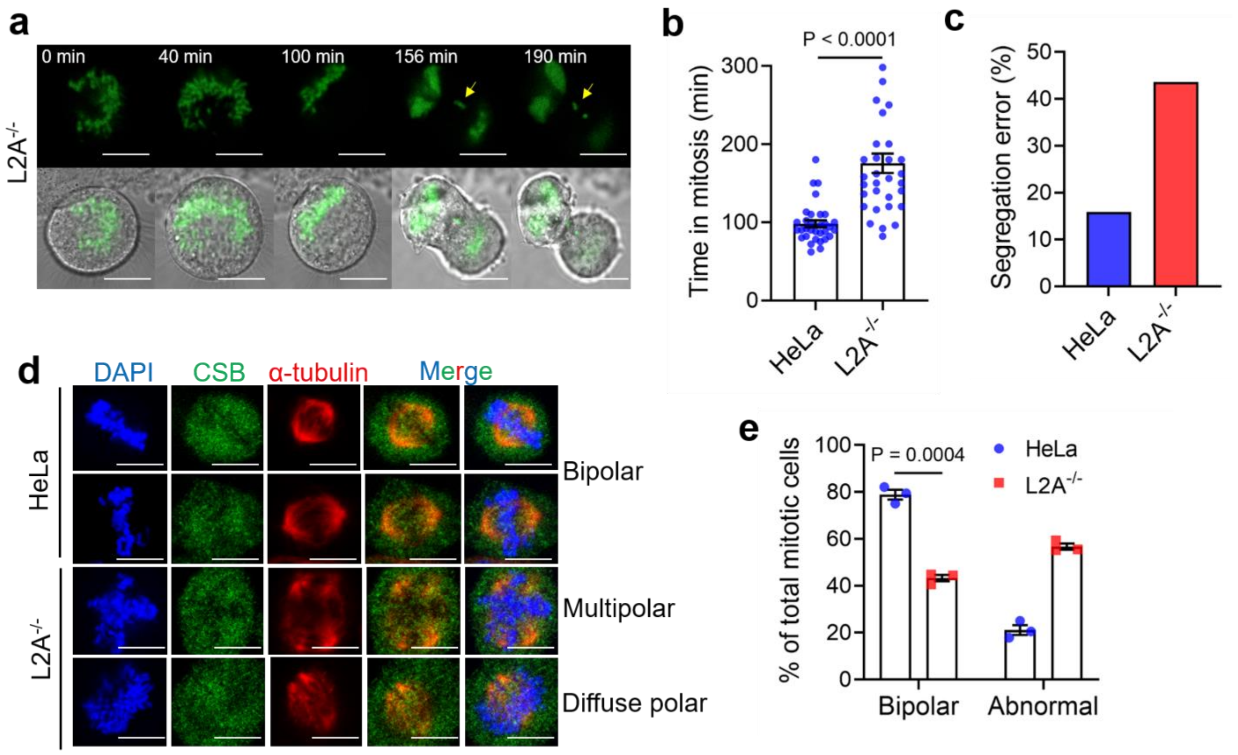

Supplementary Fig. 5: CMA-deficiency leads to aberrant mitosis. (**a**) Representative images showing time-lapsed mitotic cell division of control and *L2A*$^{-/-}$ HeLa cells. Yellow arrow indicates lagging chromosomes. (**b, c**) Quantifications of average mitosis time (*n* = 32 cells) (**b**) and chromosome segregation error rate (*n* = 44 cells) (**c**) in control and *L2A*$^{-/-}$ HeLa cells. (**d**) Representative images showing spindle morphologies (red) in control and *L2A*$^{-/-}$ HeLa cells. (**e**) Quantification of cells with bipolar and abnormal spindles in control and *L2A*$^{-/-}$ HeLa cells, *n* = 3 experimental repeats. All multipolar, monopolar and diffuse-polar spindles were counted as abnormal spindles. Scale bar, 20 μm. Data are shown as means ± SEM. P values were determined by two-tailed unpaired t test with Welch's correction. Source data are provided as a Source Data file.

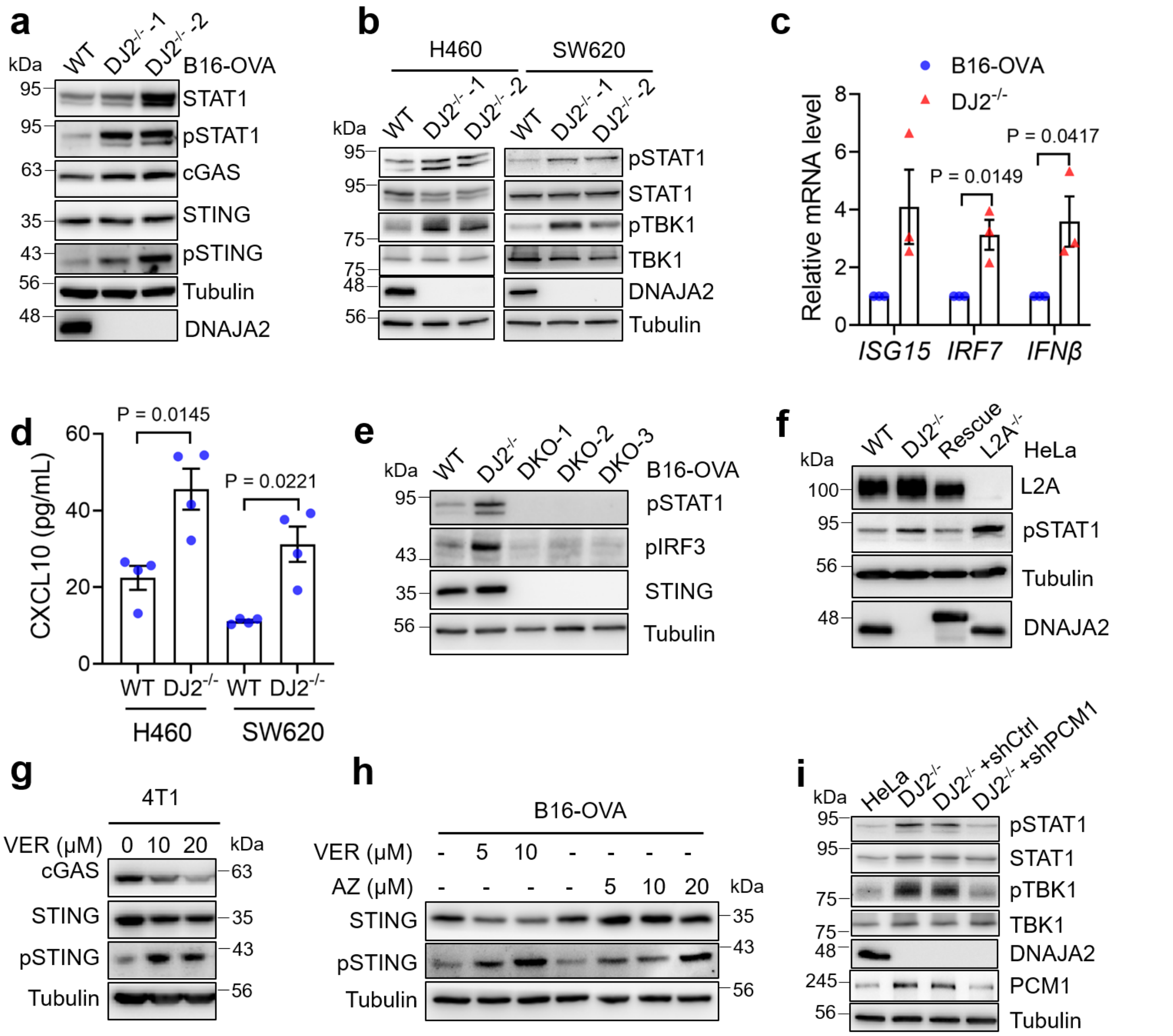

Supplementary Fig. 6: Defects in DNAJA2/HSC70-mediated CMA activate the cGAS-STING pathway. (**a**) Western blots showing expression levels of phosphorylated STAT1 (pSTAT1) and STING (pSTING) in WT and *DJ2*$^{-/-}$ B16-OVA cells, *n* = 3 experimental repeats. (**b**) Western blots showing expression levels of phosphorylated STAT1 (pSTAT1) and TBK1 (pTBK1) in WT and *DJ2*$^{-/-}$ H460 and SW620 cells, *n* = 2 experimental repeats. (**c**) RT-qPCR analysis showing relative mRNA levels of *ISG15*, *IRF7* and *IFNβ* in WT and *DJ2*$^{-/-}$ B16-OVA cells, *n* = 3. (**d**) Elisa assay showing CXCL10 level in culture media from WT and *DJ2*$^{-/-}$ H460 and SW620 cells cultured for 4 days, *n* = 4. (**e**) Western blotting analysis showing pSTING and pIRF3 in WT, *DJ2*$^{-/-}$ and *DNAJA2/STING* double knockout (DKO) B16-OVA cells, *n* = 2 experimental repeats. (**f**) Western blots showing pSTAT1 in WT, *DJ2*$^{-/-}$, *DJ2*-rescue and *L2A*$^{-/-}$ HeLa cells, *n* = 2 experimental repeats. (**g, h**) Western blots showing expression levels of pSTING in 4T1 and B16-OVA cells treated with or without VER-155008 (VER) or Apoptozole (AZ) for 24 hours, as indicated, *n* = 2 experimental

repeats. (**i**) Western blots showing expression levels of pSTAT1 andpTBK1 in WT, *DJ2*$^{-/-}$ and *DJ2*$^{-/-}$ with control shRNA (DJ2$^{-/-}$+shCtrl) or shRNA targeting *PCM1* (DJ2$^{-/-}$+shPCM1) HeLa cells, *n* = 3 experimental repeats. Data are shown as means ± SEM of *n* experimental repeats. P values in figure **c** were determined by two-tailed unpaired t test and the others were determined by two-tailed unpaired t test with Welch's correction. Source data are provided as a Source Data file.

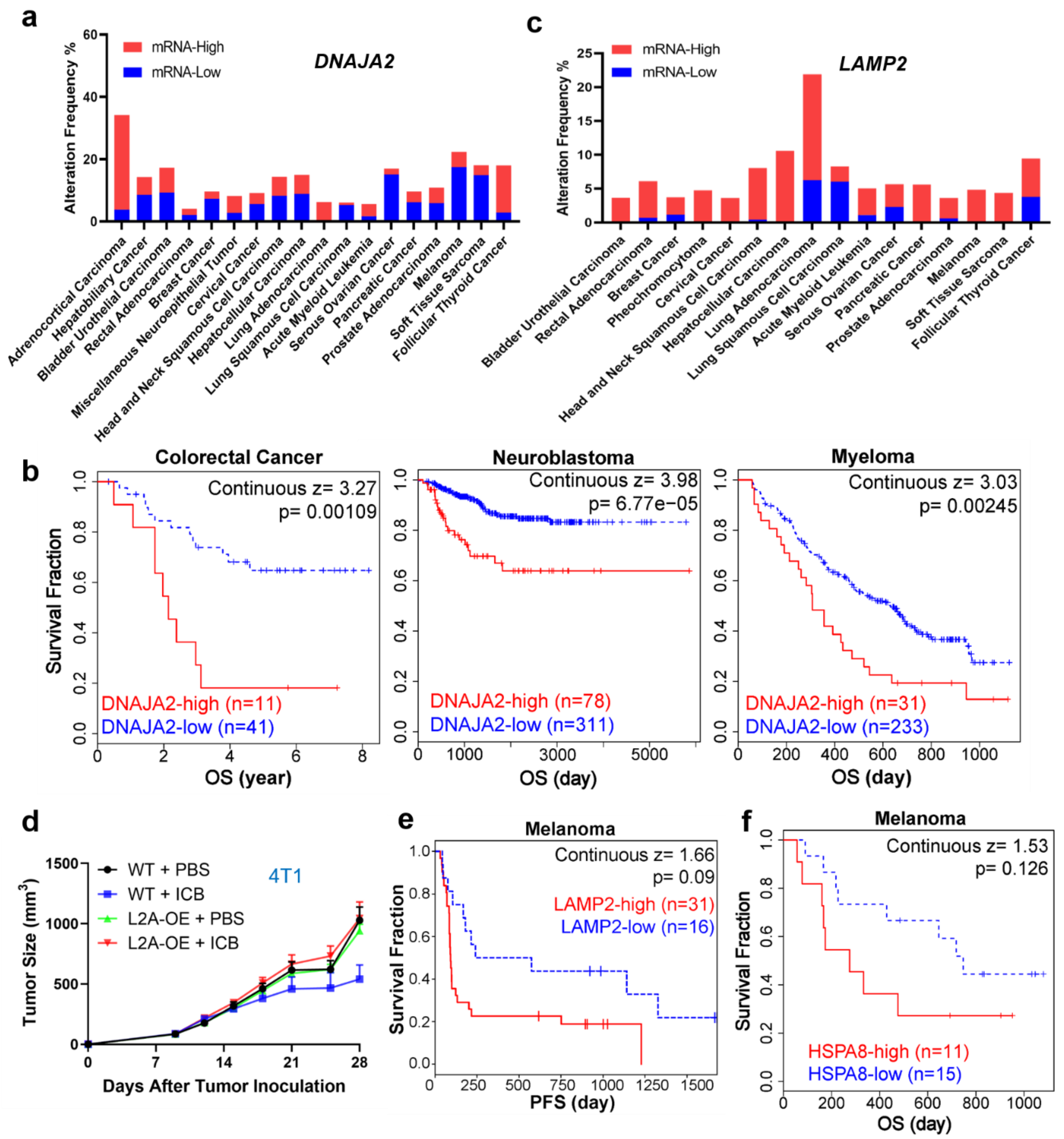

Supplementary Fig. 7: Clinical relevance of DNAJA2/HSC70-mediated CMA pathway in cancer immunotherapy. (**a**) Percentage of cancer patients with altered expression of *DNAJA2* in cBioPortal database[1,2]. (**b**) Survival curves of cancer patients with low or high expression levels of *DNAJA2* in various cancer types[3]. (**c**) Percentage of cancer patients with altered expression of *LAMP2* in cBioPortal database[1,2]. (**d**) Tumor growth curves (means ± SEM) of WT and *L2A*-overexpressing (*L2A*-OE) 4T1 tumors treated with or without ICB therapy. WT or *L2A*-OE 4T1 cells were inoculated into BALB/c mice (n = 7 mice) and treated with anti-CTLA4 and anti-PD-L1

antibodies at day 9, 12 and 15. (**e**) Progression-free survival (PFS) rate of melanoma patients with high or low expression levels of *LAMP2* in a clinical cohort treated with anti-PD1 immunotherapy[3]. (**f**) Overall survival (OS) rate of melanoma patients with high or low expression levels of *HSPA8* (coding for HSC70) in a clinical cohort treated with anti-PD1 immunotherapy[3]. P values in figures **b**, **e** and **f** were determined by Kaplan-Meier analysis. Source data are provided as a Source Data file.

1 Cerami, E. *et al.* The cBio cancer genomics portal: an open platform for exploring multidimensional cancer genomics data. *Cancer Discov* **2**, 401-404 (2012).

2 Gao, J. *et al.* Integrative analysis of complex cancer genomics and clinical profiles using the cBioPortal. *Sci Signal* **6**, pl1 (2013).

3 Fu, J. *et al.* Large-scale public data reuse to model immunotherapy response and resistance. *Genome Med* **12**, 21 (2020).
