# Supplementary material for: DNAJA2 deficiency activates cGAS-STING pathway via the induction of aberrant mitosis and chromosome instability
Source: Nat Commun. 2023 Aug 28;14:5246. doi: 10.1038/s41467-023-40952-0 (PMC10462666; doi:10.1038/s41467-023-40952-0)
Supplement: Supplementary file 2 — Description of Additional Supplementary Files [file 41467_2023_40952_MOESM2_ESM.pdf]

File Name: Supplementary Data 1
Description: Details of all the antibodies, chemicals, oligonucleotides and plasmids used in this study.

File Name: Supplementary Movie 1
Description: Live-cell video of multinuclear cell formation.

File Name: Supplementary Movie 2
Description: Live-cell video of chromosome segregation in mitotic division.

File Name: Supplementary Movie 3
Description: Live-cell video of chromosome segregation and spindle morphology in mitotic division.
