# Supplementary figures and images for: DNAJA2 deficiency activates cGAS-STING pathway via the induction of aberrant mitosis and chromosome instability
Source: Nat Commun. 2023 Aug 28;14:5246. doi: 10.1038/s41467-023-40952-0 (PMC10462666; doi:10.1038/s41467-023-40952-0)

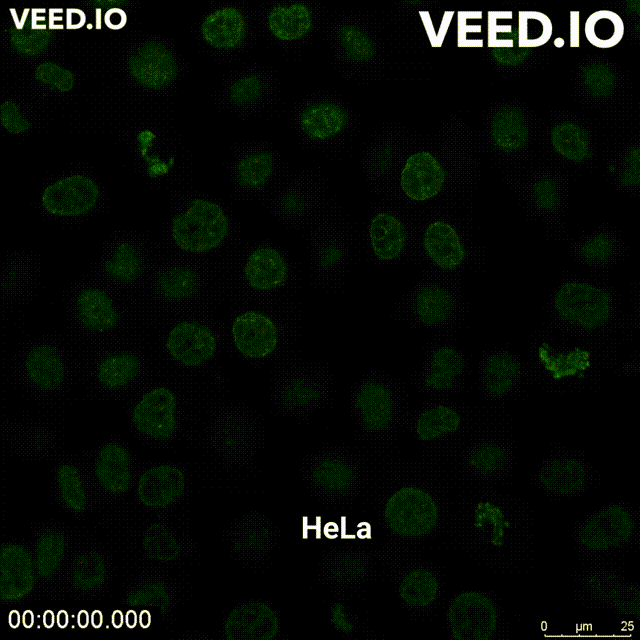

Supplement: Supplementary file 5 — Supplementary Movie 1 [file 41467_2023_40952_MOESM5_ESM.zip › Supplementary Movie 1.gif]

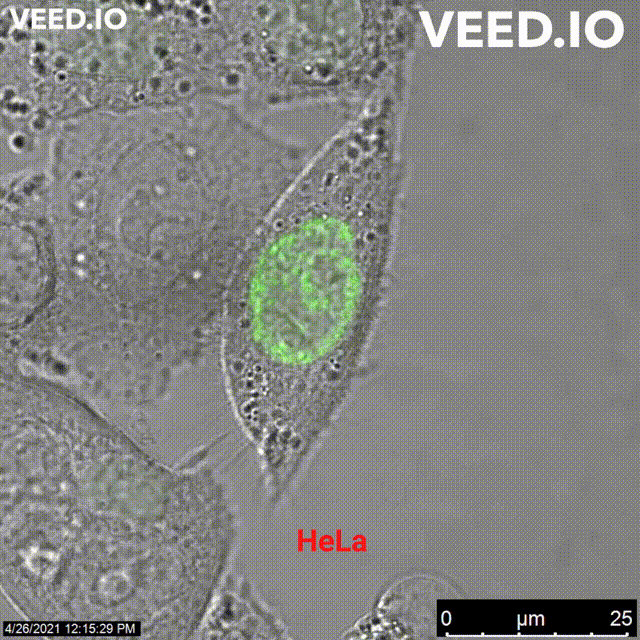

Supplement: Supplementary file 6 — Supplementary Movie 2 [file 41467_2023_40952_MOESM6_ESM.zip › Supplementary Movie 2.gif]

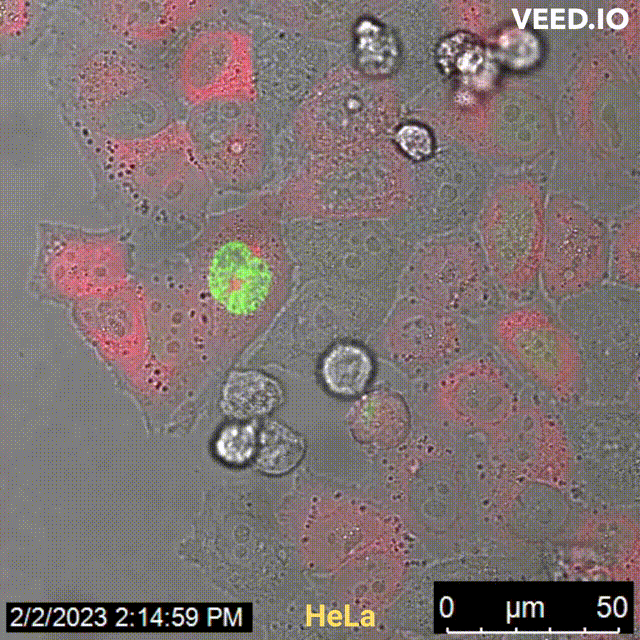

Supplement: Supplementary file 7 — Supplementary Movie 3 [file 41467_2023_40952_MOESM7_ESM.zip › Supplementary Movie 3.gif]
